# Perceptual simultaneity and its modulation during EMG-triggered motion induction with electrical muscle stimulation

**DOI:** 10.1371/journal.pone.0236497

**Published:** 2020-08-12

**Authors:** Seito Matsubara, Sohei Wakisaka, Kazuma Aoyama, Katie Seaborn, Atsushi Hiyama, Masahiko Inami

**Affiliations:** 1 Graduate School of Information Science and Technology, The University of Tokyo, Tokyo, Japan; 2 Research Center for Advanced Science and Technology, The University of Tokyo, Tokyo, Japan; 3 Virtual Reality Educational Research Center, The University of Tokyo, Tokyo, Japan; 4 Department of Industrial Engineering and Economics, Tokyo Institute of Technology, Tokyo, Japan; 5 RIKEN Center for Advanced Intelligence Project, Tokyo, Japan; Toronto Rehabilitation Institute - UHN, CANADA

## Abstract

When human movement is assisted or controlled with a muscle actuator, such as electrical muscle stimulation, a critical issue is the integration of such induced movement with the person’s motion intention and how this movement then affects their motor control. Towards achieving optimal integration and reducing feelings of artificiality and enforcement, we explored perceptual simultaneity through electrical muscle stimulation, which involved changing the interval between intentional and induced movements. We report on two experiments in which we evaluated the ranges between detection and stimulus for perceptual simultaneity achievable with an electromyography-triggered electrical muscle stimulation system. We found that the peak range was approximately 80-160 ms, with the timing of perceptual simultaneity shifting according to different adaptation states. Our results indicate that perceptual simultaneity is controllable using this adaptation strategy.

## Introduction

**Fig 1:**
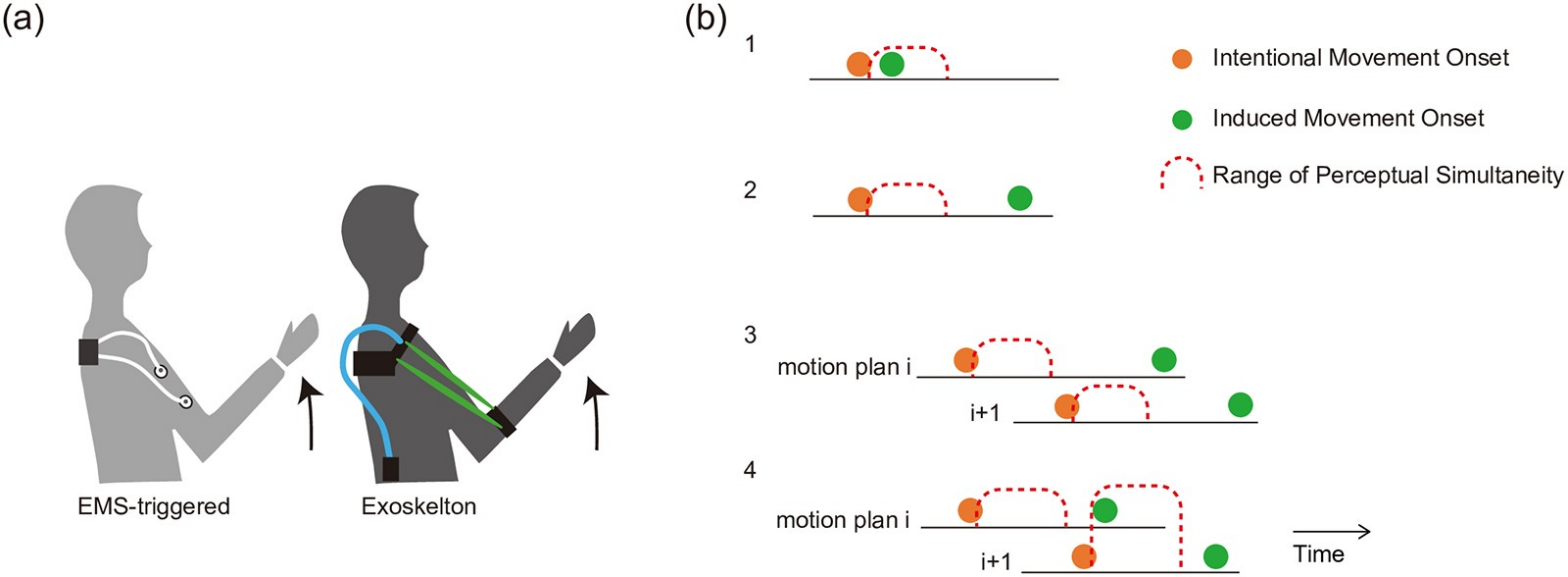
Motion induction. (a) Muscle actuation and exoskeleton actuation. Various implementations for exoskeletons have been proposed, such as McKibben artificial muscle [2, 5]. (b) Illustration of perceptual simultaneity between intentional movements and induced movements. Four conditions. Note that in this illustration the range is not precise, and will be psychophysically investigated in this paper.

Human beings have continuously developed tools to enhance quality of life. Recent technological advances have allowed for the development of various systems that can interactively assist our physical capabilities (e.g., power assist suits [1, 2], human-machine interactive tools [3, 4]). When human movement is assisted with a muscle actuator or an exoskeleton suit (Fig 1a), however, a critical issue arises: how to integrate the induced movement with the intention of the person and their motor control mechanism. This integration requires an exploration of human factors (e.g., perception of the induced movement) as well as technical factors (e.g., system latency, power control quality). For perception, timing is particularly important.

When induced motion immediately follows voluntary movement (i.e., movement at the person’s volition, or their intentional movement) in the range where perceptual simultaneity holds, it is perceived by the person as “synchronized” (Fig 1b1). When induction is beyond this range, the person perceives the movement as “late.” Late induction itself does not necessarily disrupt the motor control mechanism, so long as the correspondence between intentional and induced movement is held and it does not disturb the person’s intention to move and their actual movement (Fig 1b2). For example, in the case of a gait assist suit, when the original steps are relatively slow (e.g., 1 s), a 500 ms-delayed induced motion can still effectively support the gait motion (although the person may be aware of the delay). Now, suppose that the motion consists of a series of consecutive motion plans. In such a situation, the correspondence would be lost due to late induction, leading to the prevention of integration (Fig 1b3 and 1b4). For example, if a person intends to move their arm up slightly and then quickly to the left, the upwards movement of their arm should not be continually assisted upward. If that happens, not only is the movement disturbed, but also the person might be confused about what they originally were trying to do with their arm. Indeed, false synchronization has been a serious problem in this and related areas of research.

**Fig 2:**
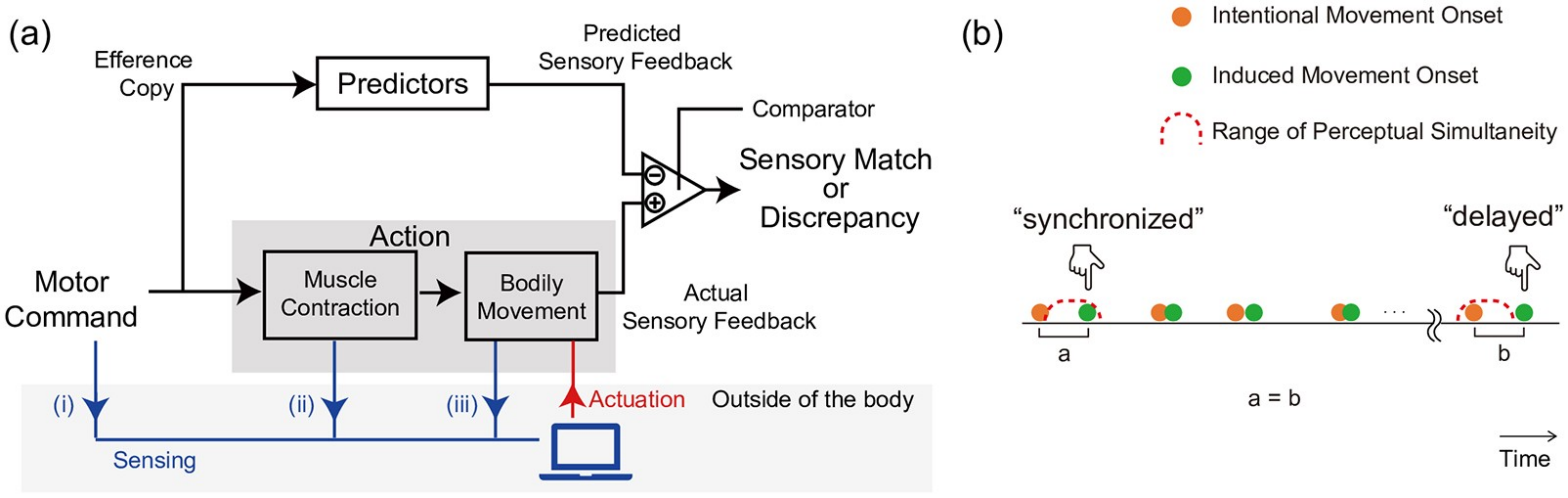
The neurocognitive comparator mechanism and our working hypothesis. (a) A simple illustration of the neurocognitive comparator mechanism with sensing and actuation (based on [7]). A forward model makes predicted sensory feedback based on an efference copy, which is an internal copy of a movement-producing signal. The comparison between predictions and actual sensory feedback results in either a sensory match or a discrepancy. A sensory discrepancy between the predicted and actual sensory feedback results in a lack of SoA and accordingly creates disturbances in motor control. The blue arrows represent external sensing processes and the red arrow represents an actuation process. Where sensing occurs on this pathway depends on the sensing method, i.e., (i) electroencephalogram, (ii) electromyogram, (iii) accelerometer. Brain activity-based sensing is earliest, followed by muscle activity sensing and then motion sensing. (b) Illustration of our working hypothesis.

This issue has been explored and modeled as a comparator model [6, 7]. In this model, a sensory match between predicted and actual sensory feedback results in a sense of agency (SoA). Furthermore, discrepancies will result in a lack of SoA and accordingly disturbances in motor control (Fig 2a). In other words, the induced movements are perceived as enforced by some external mechanism. This rests on the notion that simultaneity between intentional and induced movements is a basic and crucial factor for achieving integration. In this paper, we specifically focus on simultaneity, while also recognizing that other related factors, such as motion trajectory and actuation intensity, are crucial for achieving integration, i.e., a sensory match.

According to previous studies, perceptual simultaneity is flexible. A repetitive constant delay between two stimuli, each from a different modality (e.g., audio and visual), causes a realignment of these stimuli in perception (i.e., temporal recalibration) [8, 9]. When multiple stimuli are issued in a short span of time, the perception of these stimuli can be inhibited (i.e., through masking) [10]. Interval between voluntary actions and their subsequent outcomes are perceptually compressed (i.e., intentional binding) [11]. Although the underlying mechanism of this plasticity is still under debate, we hypothesize that a similar effect would be observed between intentional and induced movement. That is, after repetitive exposure to delayed actuation of the motion intention, the perceptual simultaneity between the intentional and induced movement can be adjusted (Fig 2b).

To study perceptual simultaneity and its modulation, we developed a motion induction system that uses electromyography (EMG) as a trigger. This system detects the onset of bodily movements using an EMG device and stimulates the biceps to induce additional movements through electrical muscle stimulation (EMS). We used EMG for several reasons. Brain activity-based sensing can predict the onset, but is not suitable for precisely identifying which body part a person intends to move. Motion sensing with accelerometers (ACCs) are stable and robust, but they only detect movements after execution. In this study, detection before execution is required for obtaining a psychophysical profile of perceptual simultaneity. This can be done by measuring muscle contractions related to movement execution with EMG. Similarly, we adopted EMS as the actuation method because it directly stimulates the peripheral nervous system and then contracts biceps before the movement onset, as in voluntary movements. (see the blue and red lines in Fig 2b).

Various studies have used a combination of EMG and EMS. Nishida and Suzuki developed an EMG and EMS device for recording EMG data during stimulation with EMS to share kinesthetic experiences [12]. In rehabilitation research, EMG-triggered functional electrical stimulation (FES) has been used to improve the motor function of patients paralyzed due to stroke. Cauraugh et al. reported on the effects of EMG-triggered EMS on the wrist and finger extension muscles [13]. Muraoka developed an EMG-modulated FES device that detects intentional movements through EMG via stimulation electrodes [14]. Hara et al. reported on the effects of this device on fingers [15]. These studies mainly focused on the rehabilitation of paralyzed patients; temporal perception was not measured or explicitly discussed.

In this research, we first explored the temporal range of perceptual simultaneity and then observed any shift of the timing of perceptual simultaneity between intentional and induced movements due to adaptation. In Experiment 1, we sought to identify the interval between detection and stimulation for subjective simultaneity. In Experiment 2, we examined whether or not perceptual simultaneity can be re-calibrated. This work contributes to our understanding of the plasticity of perceptual simultaneity.

## Research instrument

**Fig 3:**
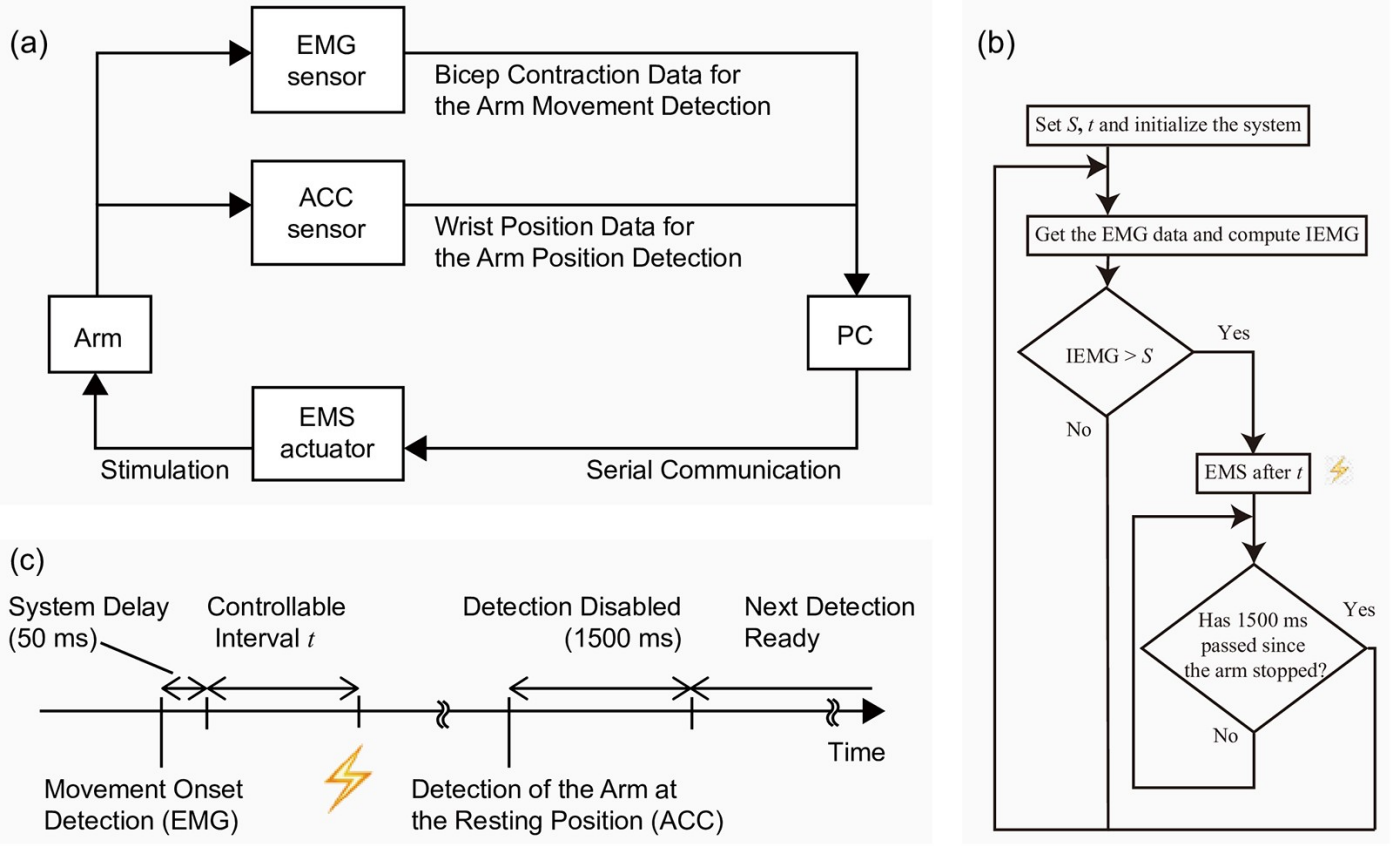
System diagram and sequence. (a) System diagram. The system detects the arm movement onset via EMG and position with ACC. (b) Detection-stimulus workflow. *S* is the detection threshold and *t* is the controllable interval. (c) Detection and stimulation sequence. The system delay between the detection and stimulation is approximately 50 ms. This total delay is controllable by inserting an additional interval. If the arm returns to a resting position and remains still for 1500 ms, subsequent EMG detection is enabled.

In this section, we describe our research instrument: the EMG-triggered motion induction system mentioned in the previous section. Fig 3a shows a diagram of the system, which has three components: (i) a detection unit, (ii) stimulation unit (EMS), and (iii) a PC with in-house software (C++). Where the sensors and actuators are placed on the human body is described in detail in the Experiment section. We selected the biceps as the detection and stimulation site, because EMS applied to the biceps results in arm bending, which is the basic gesture in related applications (e.g., lifting support).

### Detection of the movement onset through EMG

We employed a wearable body motion sensing platform (biosignalsplux), which uses EMG and ACC sensors. The sampling rate of these sensors was 1 kHz. We used ACC for detection of the motion of the arm returning to a resting position. For our purposes, we needed to detect the movement onset as soon as possible. For this reason, we used a detection algorithm with EMG, which statistically detects the onset before the actual movement. The gain of the EMG is 1000, and its raw signal ranges from −1.5 mV to 1.5 mV.

Fig 3b shows the detection-stimulus workflow of the developed system. To detect the movement onset, we used a 20-frame (20 ms) integrated EMG (IEMG) [16], which has low computational complexity. IEMG is defined as a function (1).

$$\text{IEMG}=\sum_{i=1}^{20}\left|x_{i}\right|$$ (1)

where *x*_*i*_ denotes the EMG signal in segment *i*. The system detects movements when the IEMG exceeds threshold as a function (2).

$$S=\text{max}({\text{IEMG}}_{\text{at}\_\text{resting}\_\text{state}})+\text{offset}$$ (2)

where *S* is the threshold.

Any time the arm is at a resting position for 1500 frames, S is updated to the sum of the maximum value of IEMG within the frames and a fixed offset. The offset is determined by the following procedure every time the electrodes are attached to a person: (1) tentatively set to 0.018 mV, (2) gradually increased until the arm lifting movements (free onset) are detected 15 times consecutively, without participants reporting any error detection, and (3) fixed to this value until the electrodes are detached. S is updated because IEMG can vary depending on the condition of the muscle; for example, IEMG decreases with fatigue.

If the arm is not resting, the EMG signal of the biceps is substantially higher than the threshold. After the person has returned their arm to the resting position, the system waits for 1500 ms before reading the next EMG data. Fig 3c shows the system process.

### EMS

We apply EMS after detecting the movement via EMG (with 8 mA square AC wave, 200 Hz, 1 ms pulse width, 300 ms duration). The total current is limited only to 10 mA, allowing for safe operation.

### Detection stimulus interval (DSI)

**Fig 4:**
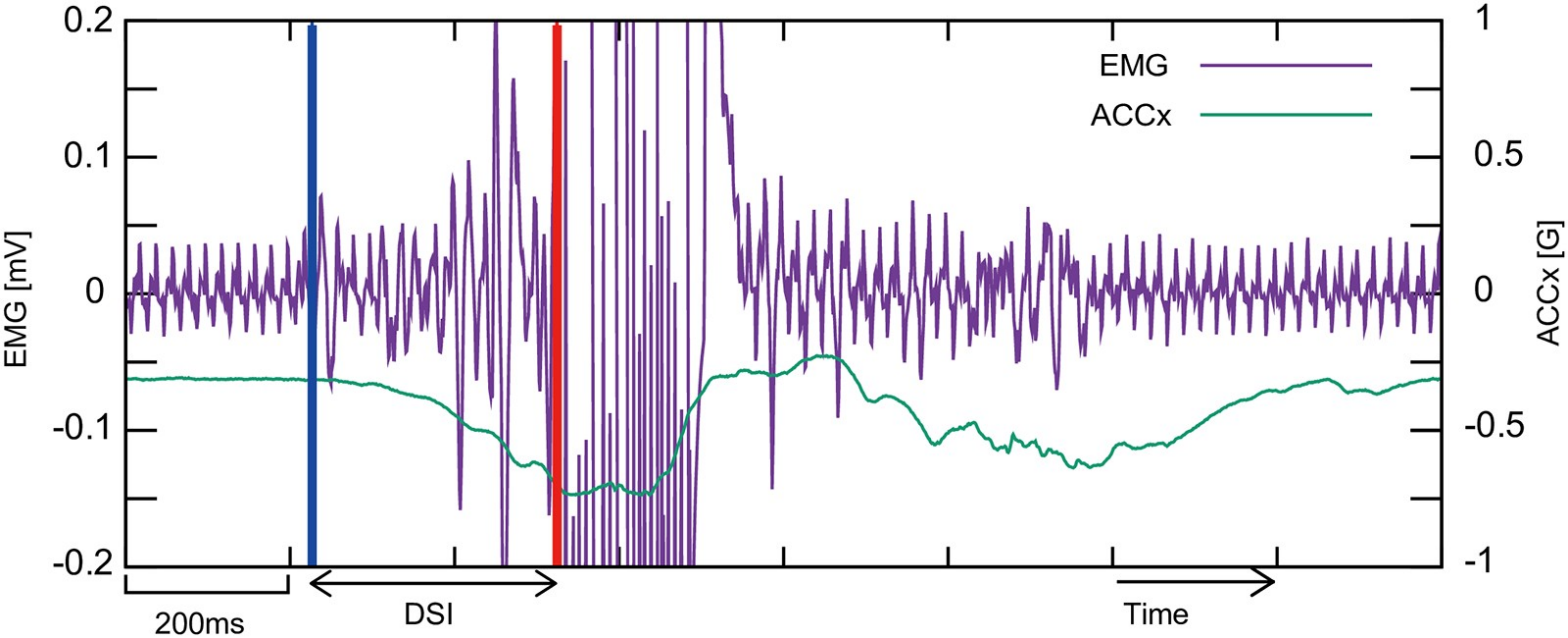
Example of the EMG and ACC signals. Blue and red lines indicate movement detection and the stimulation onset, respectively. The interval is defined as the DSI. Note that the EMG signal goes off scale after the stimulation and then returns to a stable state.

The interval between the detection and the stimulation (DSI) is controlled by our custom-made software. Fig 4 shows an example of the signals from EMG and ACC, and DSI. In the analysis, EMS timing was determined from EMG data, because it is more precise than the timing obtained from the PC, considering the system delay variation (i.e., from the detection to the EMS actuation. average: 48.3 ms, SD: 20.1 ms). The total delay is controllable by inserting an additional interval.

## Experiment 1: Temporal range of perceptual simultaneity between intentional and induced movement

In this section, we identify the range of DSI wherein simultaneity is retained. The stimulation was applied to the participants prior to or after the onset of arm movement, with randomized intervals. Then we calculated the DSI range of perceptual simultaneity based on the EMG data and subjective judgment of the simultaneity for each stimulation.

### Methods

#### Participants

Eleven healthy male adults participated in this experiment (mean age 22.0 ± 1.0 years). All experiments were approved by the Life Science Research Ethics and Safety Office at the University of Tokyo, Japan (approval number: 18-322). Moreover, all participants signed a letter of consent after provided with an overview of the experiment and instructions. The study protocol was performed in accordance with the ethical standards provided in the Declaration of Helsinki.

#### Preparation

**Fig 5:**
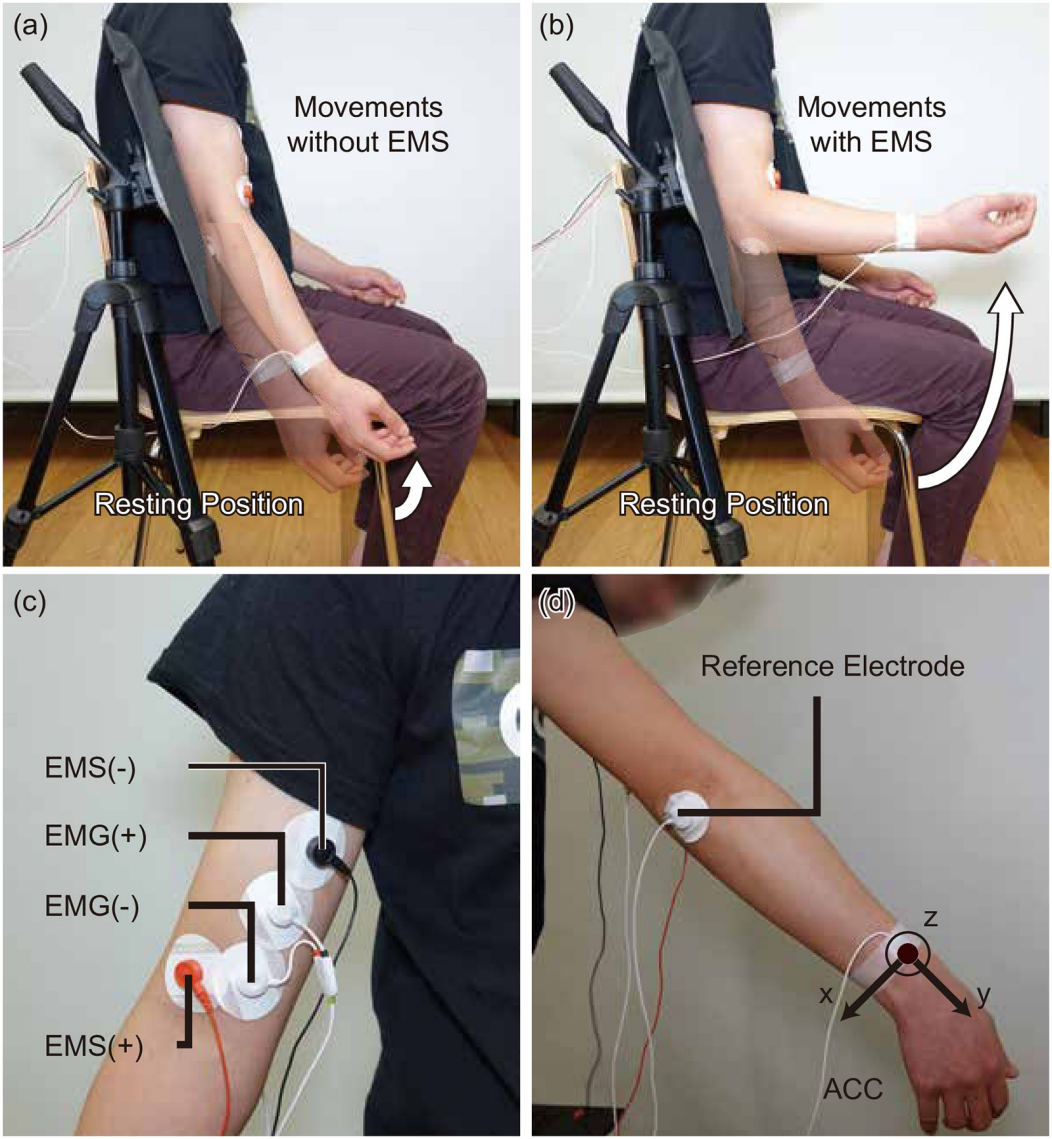
Setup of the experiments. (a) Participants are required to make small movements. (b) With EMS, the movements are amplified to larger movements. (c) and (d) show an example of where the electrodes and ACC sensor were placed.

Participants sat on a chair and placed one elbow at a resting position. EMS and EMG electrodes were attached to the biceps of the participants and an ACC sensor was attached to the wrist. Fig 5 shows the small movement that participants were instructed to make (a) and an induced movement with EMS (b). Fig 5(c) and 5(d) show the sample configuration of the electrodes and ACC. The configuration was individually adjusted to ensure that the EMS shows a visible lifting of the upper arm (approximately 30° or above).

#### Instructed timing of the movement onset, detection timing

Stimulation timing is an important feature of the trials. If the stimuli are always triggered by EMS detection, all stimulation would consist of ‘post-detection’ stimulation. However, if the distribution of stimuli are significantly biased towards post-detection, this could affect the responses of participants (i.e., participants could start guessing the population distribution of the stimuli). Therefore, the stimulation timing needs to be distributed before and after detection, to the extent that participants cannot guess the population distribution.

To achieve this, participants were required to lift their arm at timings instructed by a monitor. This procedure assured that the motion detection timing would be stochastically distributed near (before and after) the instructed timing. Consequently, applying EMS equally before and after the instructed timing, the stimulation timing would be distributed near the intended timing of motion onset by participants. The resulting detection-instruction interval histogram and the detection-stimulation interval histogram will be verified in the Results section. Note that, for our purposes, the distribution did not have to be precisely symmetric. Note also that only those trials with post-detection stimulation were used in the DSI-based analysis.

#### Training session

EMS was applied for 10 s to habituate participants to the stimulus and prevent a startle reaction. Then, the participants were stimulated immediately after the detection of voluntary movement 100 times repetitively, without instruction about movement timing. In those routines, the DSI was at minimum, i.e., a system delay of only (48.3 ms, SD = 20.1 ms). Next, participants practiced moving their arms at a certain timing, which is required in the main task. Countdown digits were displayed on the monitor (i.e., “3”, “2”, “1”, “0”); participants were instructed to move their right arm when “0” was displayed. This process was repeated until they successfully moved their arm within the range of ± 100 ms for five times consecutively.

#### Task

**Fig 6:**
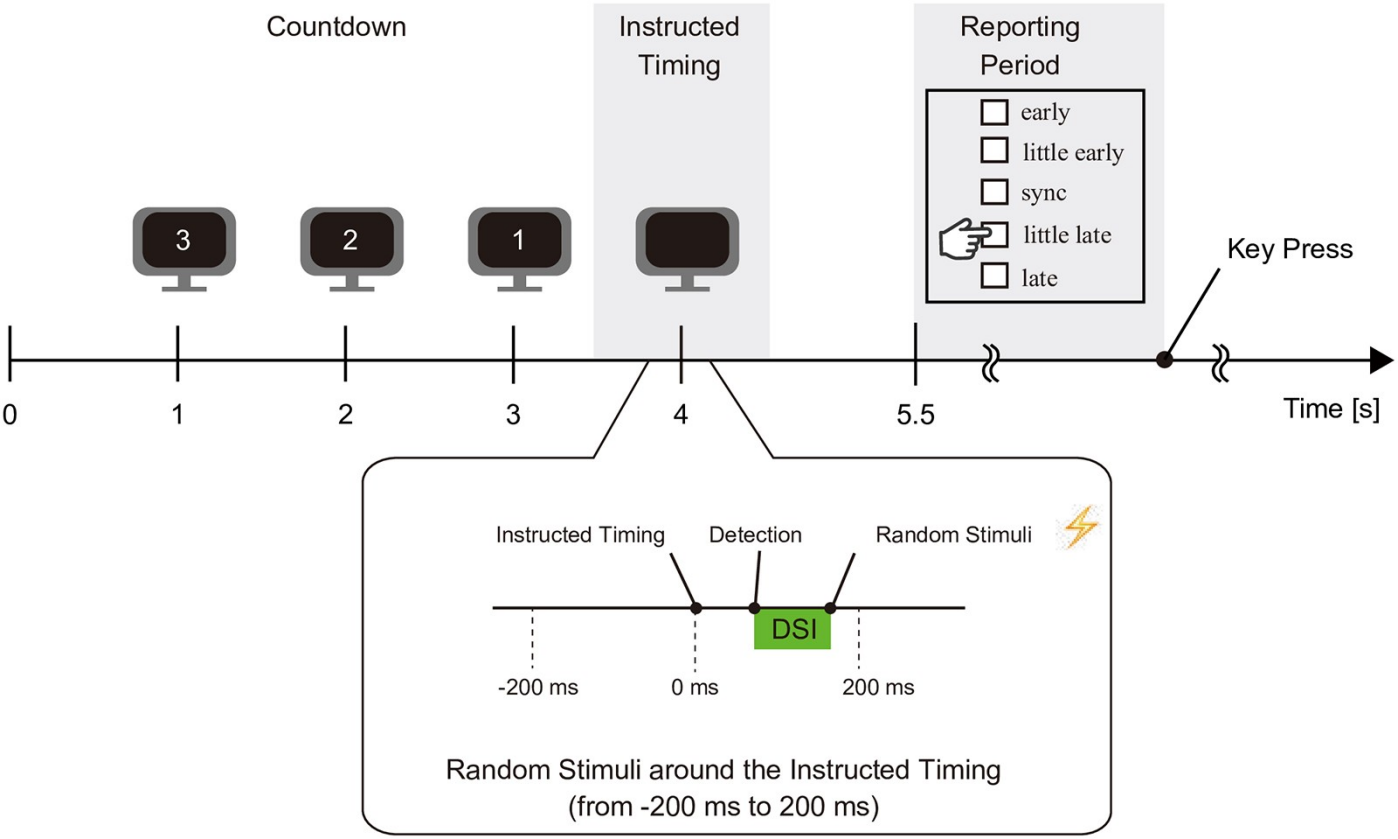
Timeline of Experiment 1. The timing of the random stimuli varies for each trial. To get participants to focus only on their movement intention and the timing of the EMS, the “0” digit was not presented. EMS was induced with a delay randomly chosen from a set of 11 values between −200 ms and +200 ms, before and after the instructed timing.

Fig 6 shows the timeline of this experiment. As in the training session, countdown digits were displayed, but the “0” was not shown (i.e., “3”, “2”, “1”). Instead, participants were instructed to move their right arm at what they perceived to be time zero (i.e., “instructed timing”). We did not present a number at time zero because, if displayed, it could act as a baseline in the simultaneity judgment task. We could not allow this to occur because the simultaneity of concern is between the intended timing of movement onset and EMS timing, not between the number display timing and EMS timing. However, by the nature of the experimental design, inhibition is practically impossible. Therefore, to get participants to focus only on their intention (not the displayed number) and the timing of the EMS, the “zero” number was not presented.

EMS was induced with a delay randomly chosen from 11 values between −200 ms and +200 ms, before and after the instructed timing. Assuming that the actual arm movement timing remained as accurate as in the training session, about half of the stimuli were supposed to be applied prior to detection. This symmetrical stimulus distribution is necessary for statistical analysis and is verified in the results section.

In addition, participants were asked to report when the stimulus-based movement was performed compared to the movement they carried out (or planned to carry out) by their own volition. They reported this in five stages (i.e., early, a little early, synced, a little late, and late) by pressing the corresponding keyboard button with their left hand.

#### Preprocessing and data analysis

All data were analyzed with R (Version 3.5.3).

Trials that did not satisfy two criteria for DSI-based analysis were excluded. The criteria were: (i) no detection before stimulation and (ii) false positives.

For (i), trials where stimulation without detection occurred were excluded from analysis. The DSI cannot be calculated in such cases. In these cases, the stimulus is considered to be applied before motion intention. We expected about half of all trials would be excluded based on this criterion. Indeed, 429 out of 1100 trials were excluded.

For (ii), data in which the onset detections by EMG and ACC were separated by a certain amount of time were excluded. The duration was determined using the Hampel identifier, an outlier detection algorithm [17]. The algorithm excluded the data out of the range of [-133, 233]. The exclusion range is consistent with previous works on the EMG detection timing and the movement timing [18, 19]. In these cases, the EMG detections were thought to be false positives (i.e., no actual movement occurred after detection by EMG). In the end, 109 out of 671 trials were excluded.

Additionally, one participant’s data were also excluded because their “a little late” and “late” responses were not reported at all; we concluded that the experimental instructions had not been understood. Finally, “early” and “a little early” responses were combined, as were the “late” and “a little late” responses, because the “late” and “early” responses were much fewer in number (approximately 5% of all data) compared to the other responses.

We calculated the point of subjective equality (PSE) and just noticeable difference (JND) to identify the DSI range of perceptual simultaneity statistically. The cumulative normal distribution functions were fit to the data using the maximum likelihood method to calculate PSE and JND. The PSE is defined as the mean of the cumulative normal distribution function, indicating the interpolated 50% crossover point. The JND is calculated by multiplying the standard deviation of the fitted cumulative normal distribution with a Zscore of probability of 0.75 (approximately 0.6745), indicating the distance between any two X-axis values where the function crosses 0.50 and 0.75 (or 0.25 and 0.50) [20]. A shift in PSE indicates the time during which people feel perceptual simultaneity has been changed. A higher JND (i.e., a steeper curve) indicates that the discrimination task was relatively easier.

### Results

**Fig 7:**
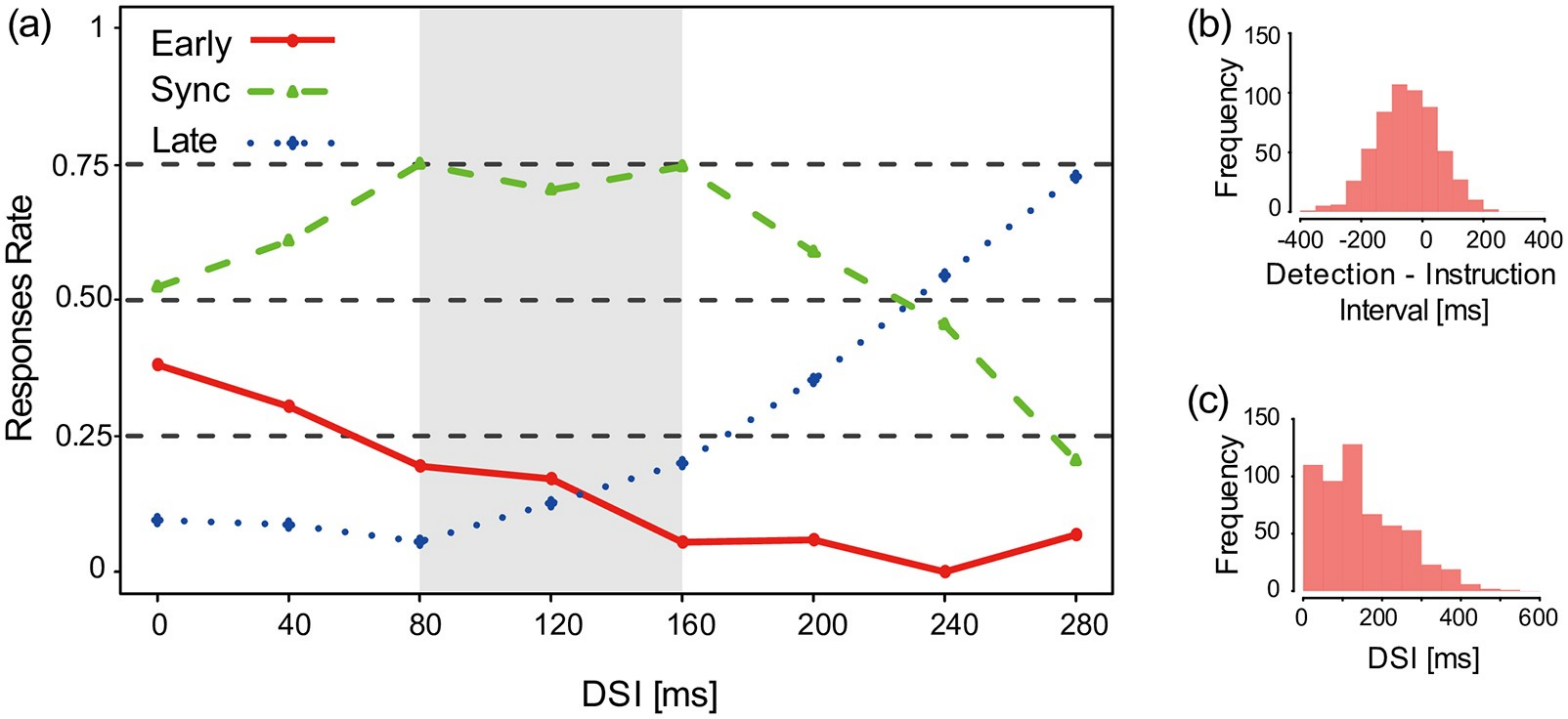
Results in Experiment 1 (n = 10). (a) Grand average of response rates in Experiment 1 (n = 10). Each point indicates the middle of a bin of ±20 ms. The sync rate reached a peak where the DSI was approximately 80–160 ms. The early and late rates intersected near 130 ms (between the “120 ms” and “160 ms” bins). (b) The distribution of detection timing towards instructed timing. The onset detection timings did not deviate from the instructed timing significantly. (c) The resulting DSI histogram. All data consists of trials with stimulation after detection.

We first confirmed that the stimuli were distributed properly before and after the instructed timing (Fig 7b). Fig 7c illustrates the resulting DSI histogram. All data consists of trials with stimulation after detection. Note that the distribution is continuous, as expected. Fig 7a illustrates the transition of the response rate against DSI (n = 10). All data from all subjects were combined together instead of using the average of individual rates. The DSI was binned at ±20 ms (40 ms range). The sync rate reached a peak where the DSI was approximately 80–160 ms. The early and late rates intersected near 130 ms (between the “120 ms” and “160 ms” bins).

**Fig 8:**
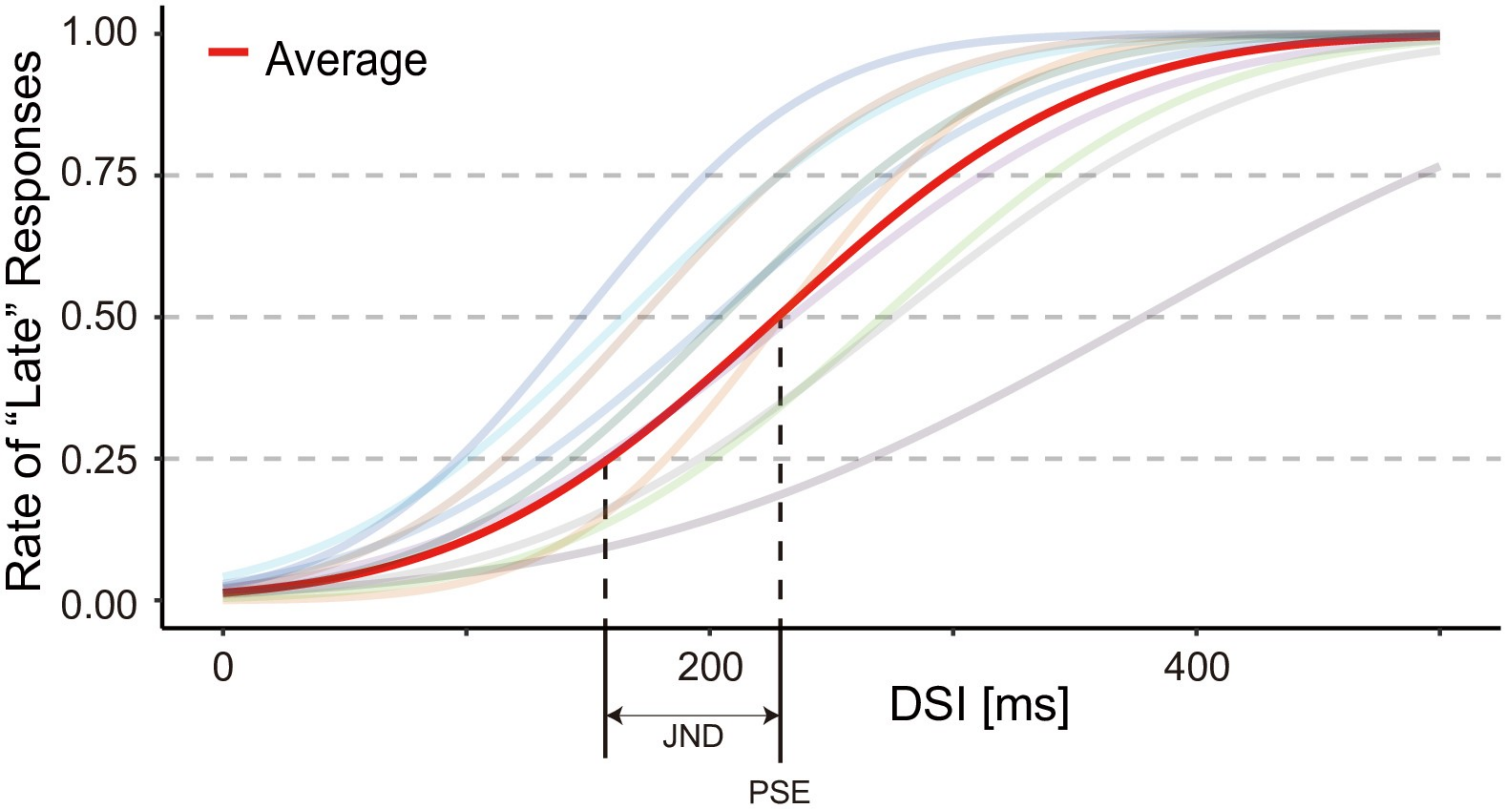
Response rates in Experiment 1. Data were fit with a probit function to capture the rate of “late” responses to DSI in the task. The crossover point of each line and the horizontal line at 50% rate of “late” responses were taken as the point of subjective equality (PSE). The just noticeable difference (JND) indicates the distance between any two X-axis values where the function crosses 0.50 and 0.75 (or 0.25 and 0.50). The average PSE and JND between “early/sync” and “late” were 228 ms and 69 ms, respectively.

Next, to evaluate the range statistically, we calculated the PSE and JND. Since a given response consisted of three alternatives, two cumulative normal distribution curves could be calculated (i.e., the curve between “early” and “sync/late” response and the curve between “early/sync” and “late”), depending on the data distribution. In our case, the former curves could not be calculated due to a significant lack of early responses, while the latter curve could be calculated. Therefore, we interpreted the results based on the latter curves. The PSE and the JND between early/sync and late were 228 ms and 69 ms (Fig 8). The twenty-five percent line of late responses was 159 ms DSI (i.e., PSE-JND). The PSE-JND in Fig 8 corresponds to the upper boundary of the DSI range of perceptual simultaneity (Fig 7a). Note that we observed individual differences in PSE. We concluded that a DSI of approximately 80-160 ms corresponds to the range of perceptual simultaneity, in general.

Following this, we needed to consider how robust the range of perceptual simultaneity is and how stable individual differences are. Adjustability is an important factor in practical applications that use motion induction. Therefore, we next explored whether the DSI range we discovered is experimentally adjustable or not.

## Experiment 2: Perceptual simultaneity shift between intention detection and induction

In this experiment, we sought to verify the perceptual simultaneity shift with our motion induction system that results from adaptation to stimuli via a biased DSI distribution. In addition to the random stimuli around the instructed timing (Stim R) that we used in Experiment 1, we introduced two new stimuli to induce adaptation. The first was stimulation with the minimum DSI, namely, system delay only (mean = 55 ms, SD = 53 ms). The second was with the system delay plus an additional interval (100 ms, total mean = 167 ms, SD = 66 ms) (Stim A50 and Stim A150, respectively). Participants were exposed to both stimuli repetitively. The perceptual simultaneity shift was defined as the shift of the DSI range of perceptual simultaneity between the two conditions. Additionally, the influence of the adaptation stimuli for pre-detection stimulation trials were also investigated.

### Methods

#### Participants

Twelve healthy adults participated in this experiment (1 woman, 11 men, mean age 25.8 ± 6.4). All experiments complied with the ethical and safety standards described in Experiment 1.

#### Preparation

The same procedure as in the training session was conducted.

#### Training session

In Experiment 1, we implemented timing synchronization using text instruction read on a display. After Experiment 1, we tested the timing instruction using beeping sounds heard through headphones. We confirmed that the beeping sounds required less cognitive load than digits displayed on a monitor. Therefore, in Experiment 2, we used beeping sounds for the timing instruction.

#### Task

**Fig 9:**
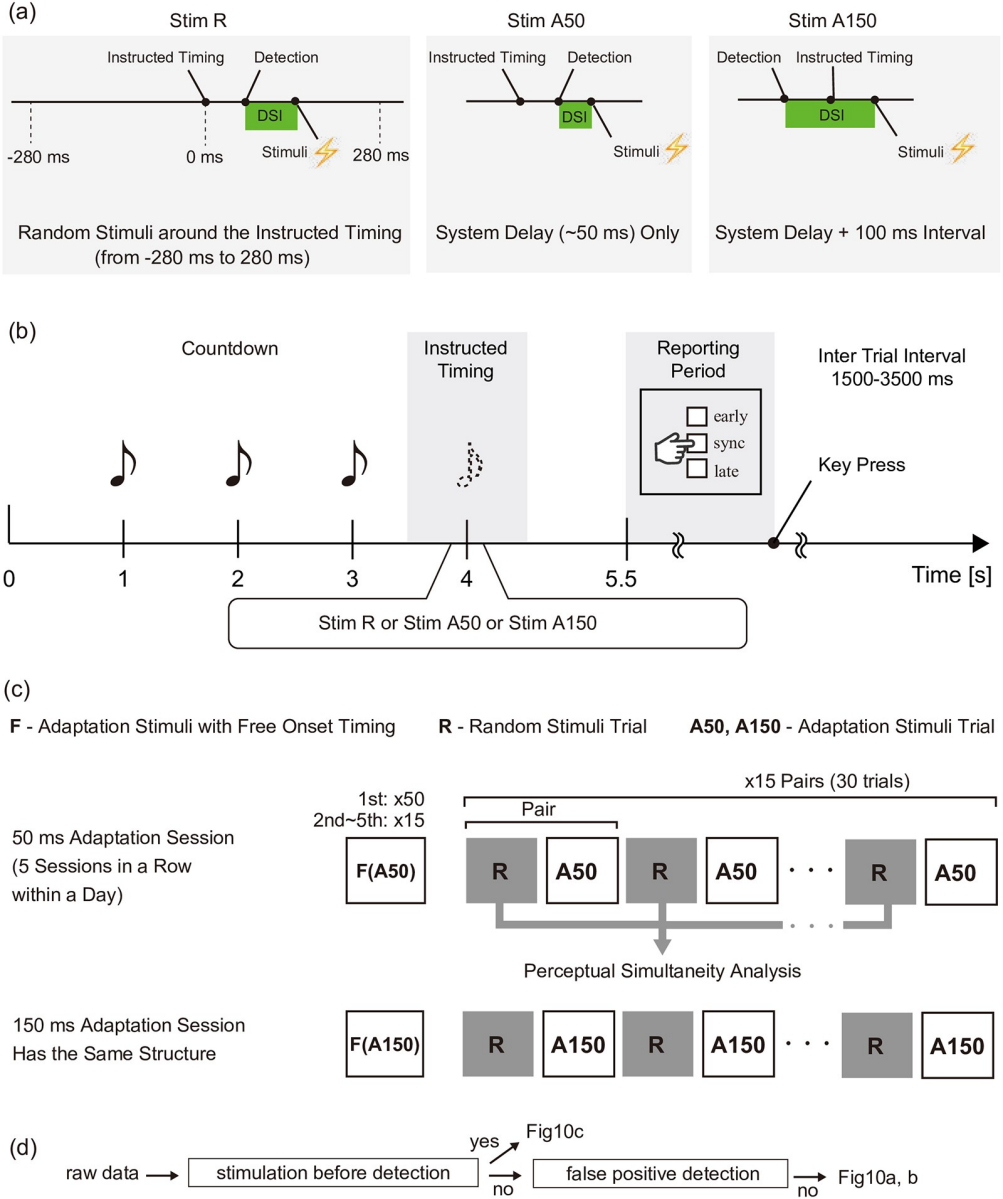
Design of Experiment 2. (a) Three types of stimuli. Note that the detection and stimuli timings vary and different in each trial. (b) The trial structure is the same as in Experiment 1, except that the countdown method was changed to beeping sounds and the simultaneity report was simplified to three scales. (c) Session structure. (d) The data flow of the analysis.

Fig 9b shows the timeline of a trial in Experiment 2. Here we introduced Stim A50 and Stim A150 trials to induce and maintain adaptation. We also used Stim R, which is identical to the Stimulus in Experiment 1, except for the range being changed to [-280 280] (Fig 9a). In the Stim A50 trials, we applied stimulation immediately after detection. Note that the resulting DSI varied due to the variation of the system delay (mean = 55 ms, SD = 53 ms). In the Stim A150 trials, we applied stimulation about 150 ms after the detection (mean = 167 ms, SD = 66 ms). The distribution of the delay is provided in the supporting information (S1 Fig). In summary, 91% of the system delays in Stim A50 were under 100 ms, indicating that the distributional separation required in this experiment was achieved.

In all trials, a beeping sound was presented three times. As in the training session, participants were instructed to move their right arm in time with when they would hear the fourth beep; note that the fourth beep was not actually played, for the same reason as digit zero was not displayed in the first experiment.

Participants were asked to report on perceptual simultaneity following a procedure identical to that of Experiment 1, except for the alternatives being changed to three stages (namely, early, sync, and late) and the button being changed to the Nintendo Wiimote controller. The main task consisted of ten 50 ms adaptation sessions and ten 150 ms adaptation sessions (Fig 9c). In the 50 ms adaptation sessions, Stim A50 with free onset timing was presented 15 times consecutively (50 times for the first session in a day). We expected that this stimuli sequence would induce adaptation. Next, trials with Stim R and those with Stim A50 were presented alternately. Stim R trials were used in the analysis of perceptual simultaneity, while Stim A50 trials were expected to maintain the participant’s adaptation status. The same procedure was applied to the 150 ms adaptation session.

The experiment was conducted over four days, and on each day only one type of adaptation session was run (i.e., two A50 ms adaptation session days and two A150 ms adaptation session days). The order of the adaptation session days was randomized.

#### Preprocessing and data analysis

All data were analyzed with R (Version 3.5.3). For assessing statistical significance, the Wilcoxon signed-rank test, a non-parametric test was used due to the small sample size (n = 12). The statistical significance (*α*) was determined using a two-sided p-value of ≤ 0.05.

With the “no detection before stimulation” criterion, the data from Stim R trials were separated into post-detection stimulation data and pre-detection stimulation data. The DSI-based analysis was applied to the data with post-detection stimulation. Specifically, the PSE and JND values in each adaptation condition were derived from the cumulative normal distribution function as in Experiment 1. This was to determine the perceptual simultaneity shift after the “false positive detection” exclusion criterion was applied.

In addition to the DSI-based analysis, we ran another analysis with the pre-detection stimulation data: comparison of the overall responses to pre-detection stimulation between the two conditions, which was not DSI-based (Fig 9d). If the responses were significantly different, it would imply that the adaptation stimuli sequences influenced perception earlier than the detection timing, and possibly even before the motion intention timing. This analysis was premised on the distributions of the detection-instruction intervals not being statistically different from the adaptation conditions. This is analyzed in the Results section.

In the end, 1820 out of 3600 trials were identified as post-detection stimulation and 320 out of 1820 trials were excluded as false positives; the data out of the range of [-128, 156] (the interval between EMG and ACC detection) were excluded.

### Results

**Fig 10:**
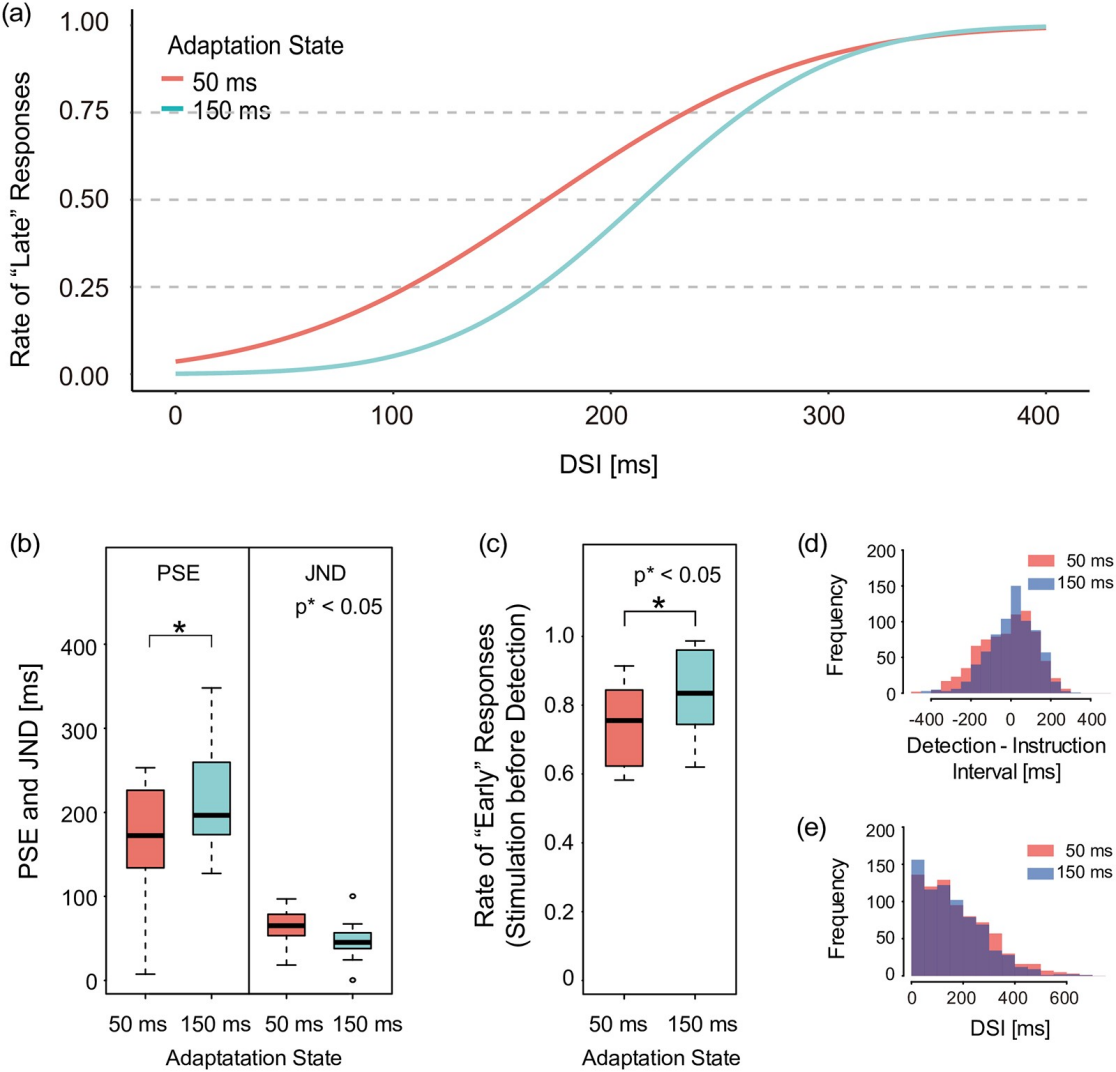
Results of Experiment 2. (a) Responses in Experiment 2 (n = 12). (b) PSE and JND means in Experiment 2. The box is drawn from Q1 to Q3 with a horizontal line used in the middle to denote the median. The PSE obtained from the 50 ms adaptation sessions was significantly smaller than that of the 150 ms sessions. (c) The rate of early responses when the stimulation was followed by detection. (d) The distribution of detection-instruction interval. (e) The resulting DSI histogram. All data consists of trials with stimulation after detection.

A raster plot of the raw data is provided in the supporting information (S1 Fig). As described in “Instructed timing of the movement onset, detection timing” subsection, the stimulation timings were required to be symmetrical to the instructed timing, which was confirmed in Fig 10d. Fig 10e shows the resulting DSI histogram. All data consists of trials with stimulation after detection. Fig 10a shows the mean responses for 50 ms adaptation sessions and 150 ms adaptation sessions, respectively. Importantly, in ten out of twelve subjects, the PSE of the latter was greater than that of the former.

The median PSEs of the adapted 50 and 150 ms DSI were 172 ms and 196 ms, respectively. They were found to be significantly different (W = 5, Z = -2.67, p = 0.0049, r = 0.576, 1-*β* (power) = 0.87, Fig 10b). The median JND of 50 ms and 150 ms adaptation sessions were 65 ms and 45 ms, respectively, and were not significantly different (Fig 10b), meaning that the shift of perceptual simultaneity occurred while the difficulty of the tasks were at the same level.

Next, we analyzed the pre-detection stimulation data. The median rates of early responses for the 50 and 150 ms adaptation sessions were 76 and 83%, respectively. They were found to be significantly different (W = 9, Z = -2.35, p = 0.016, r = 0.45, 1-*β* = 0.76, Fig 10c), while the distribution of the detection-instruction interval was not significantly different (i.e., 1 ± 27, 21 ± 22 respectively, mean ± standard error; Fig 10d). This result implies that the adaptation stimuli also influenced perception before detection.

## Discussion

In this section, we discuss the nature and mechanisms of perceptual simultaneity as illuminated by our findings and previous research. We also discuss the limitations of the system and future work based on the adaptation effects we discovered.

### Integration of induced movements with the intentions of the person

Our main finding is that integration of induced movements with intentional movements can be achieved during EMS-triggered motion induction with a DSI of approximately 80-160 ms. Furthermore, we have provided evidence that perceptual simultaneity can be adjusted experimentally. For participants who perceive simultaneity with relatively shorter DSI, we can apply longer DSI to adjust the simultaneity, and vice versa. This finding suggests that, with a carefully sequenced series of stimuli, induced movements can be integrated into our innate motor control mechanism. This can occur in spite of individual variation in the range of perceptual simultaneity.

The next and possibly most important question is: what for? Consider the situation in which motion induction supports a person’s own movements (e.g., [21, 22]). And suppose that they feel forced to move against their personal intentions in that moment, leading to feelings of discomfort and uneasiness. In such situations, we need to calibrate the actuation timing to reduce such negative feelings. Our findings are a first step towards the establishment of such a calibration method. However, in our research, we explicitly required participants to report their timing perception every time their arm moved. As such, we need to introduce a simultaneity perception test in such a way not to disturb motion plans of the participants. This is one of the most important next steps in our research, in terms of practical applications.

### Consistency between perceptions with and without adaptation

In Experiments 1 and 2, we investigated perceptual simultaneity without and with adaptation, respectively. Each PSE of the early/sync and late curves were 228 (in Experiment 1), 196 (in the 150 ms adaptation sessions), and 172 (in the 50 ms adaptation sessions). The PSE tended to be faster in the adaptation conditions than in the no adaptation condition. We cannot conduct paired statistical analysis between Experiment 1 and 2 because we recruited different participants. Even so, the PSE in the 50 ms adaptation condition was apparently lower than that in the no adaptation condition (i.e., Experiment 1), indicating that the results of these experiments are consistent with each other.

In this work, we did not apply adaptation stimuli with DSI that is smaller or exceeds the range of perceptual simultaneity. Note that such stimuli can also work as adaptation stimuli, although they may not be perceived as “synchronized” on their own. In addition, in this paper we did not examine whether stimulation before/without detection works as adaptation stimuli or not. In future research, we will explore the potentials in range of adaptation using such stimuli.

### Mechanism of the perceptual simultaneity

Variations of simultaneity shifts in perception have been studied elsewhere. The underlying mechanism of the simultaneity shift is still under debate [8, 9, 23, 24]. Some varieties may share common mechanisms and some may not. We list several factors that are thought to influence perceptual simultaneity below.

#### Temporal recalibration

Numerous studies have demonstrated that repetitive exposure to a delay between audio and visual stimuli results in the perceptual realignment of the stimuli [8, 9]. This phenomenon is referred to as “temporal recalibration” and has been observed across different modalities, including self-movement, such as visuomotor [23] and vocalization [24].

#### Intentional binding

Haggard et al. reported that voluntary actions, but not involuntary movements, are perceived to shift in time toward their subsequent outcomes and that these outcomes are perceived to be shifted toward the voluntary actions that caused them [11]. Temporal recalibration and intentional binding apparently overlap phenomenologically. Given that our setup satisfies the conditions needed to elicit these phenomena, we believe that perceptual simultaneity and its shift are likely to influenced by them. However, since the action and the subsequent outcomes are both movements in our setup, other phenomena may play a role in inducing a sense of simultaneity (e.g., backward masking, as described below).

#### Backward masking

Backward masking is a postdictive illusion where people cannot detect a weak stimulus when a strong stimulus is applied soon after the weak one [10]. In our system design, intentional movements could be masked by induced movements, affecting the psychophysical profile of the simultaneity.

### Characteristics of stimuli for adaptation

To generate adaptation states, to what extent do adaptation stimuli need to be precise? Adaptation processes during integration of motion intention (or mental imagery of motion) and motion-inducing stimulation has gained a lot of attention recently, especially in the context of neurorehabilitation. We might consider that the adaptation effect observed in Experiment 2 shares a common mechanism with the adaptation reported in previous research. Specifically, the relative timing between voluntary movements and stimulation does not need to be precise to promote neural adaptations (i.e., with spike-timing dependent plasticity) [25]. We also found that the adaptation effect in Experiment 2 is no exception; the relative timing of the adaptation stimulus (A50 and A150) was not precise due to the hardware used (see the DSI histogram shown in Supplemental Fig 1). In addition, every second stimuli (Stim R) were not biased in terms of the distribution of DSI scores. Notably, even with such variation in stimulation timing, an adaptation effect was observed. This robustness is advantageous for practical applications.

### Limitations

#### Participants

All tests were performed with healthy, young, non-disabled participants, and mostly men. As such, we can not be certain how our findings generalize to other demographics. We need to recruit more diverse participants, such as women, older adults, and people with disabilities, to see the extent to which our findings generalize.

We confirmed that the number of participants was sufficient through a post-hoc power analysis of the results of PSE (Fig 10b). However, because of the lower statistical power (1- *β* = 0.76), we must consider the analysis of the stimulation preceding detection (Fig 10c) as preliminary.

#### Technical extensions

There is a trade-off between detection accuracy and its latency, with the length of the EMG data frame as a parameter. Since the system delay is smaller than the lower boundary of the DSI range of perceptual simultaneity (as confirmed in Experiment 1), we can apply a longer data frame to enhance detection accuracy while stimulation remains within the DSI range of perceptual simultaneity. This could have several positive effects. For instance, using our algorithm, which focused on quick detection, we obtained about 18% false positives. This number could be significantly reduced with a longer data frame.

Small movements, such as twitching caused by straining oneself, can be also eliminated by using a longer data frame. For the same reason, the EMG sensing mechanism could be replaced with ACC. Indeed, ACC-based detection could be a practical solution, considering that the target muscle is not always relaxed. As for actuation, we employed EMS to explore temporal perception because it can execute fast, impulsive movements that directly intervene with the motor control system. Other solutions, such as an exoskeleton, may be applicable when the timing synchronization is not critical. (i.e., the walking support).

### Future work

#### Research trajectories

*Induced movement without motion detection*: In this study, we explored the simultaneity of induced movements applied hundreds of milliseconds before or after a person’s intention to move. But whether or not perceptual simultaneity with induced movement can exist without a priori motion detection remains an important question. If such a phenomenon is found to exist, it would mean that motion intention is postdictively generated after movement. We speculate that this is the case, because we observed several participants reporting that they were confused about their motion intention when subjected to involuntary movements. This idea will be explored in future research.

*Site, intensity, motion trajectory, action preparation*: We only studied motion induction on the biceps with relatively weak EMS intensity. As such, the profile of perceptual simultaneity is likely to change for other muscle groups and with different actuation intensities. Motion trajectory is also a key factor. The consistency between an originally intended trajectory and an induced one (e.g., via EMS) may influence the profile. In this paper, in all trials, action preparation was always accompanied (i.e., participants always intended to move their arm at the instructed timing). The existence or absence of the action preparation would also affect the perceptual simultaneity. To discover a general profile of perceptual simultaneity, we will need to explore these factors thoroughly.

*Implications for prosthetics research*: Recently, integration of real-time sensing and actuation in prosthetic devices has gained a lot of attention. To integrate such devices into an already-existing body schema and motor control system, differences in perceptions between prosthetics and intact limbs need to be thoroughly studied [26] (for the rubber hand illusion research, see [27]). Our paper is in line with this trajectory of work, studying the profile of the integration between intact limbs and motion augmentation from an external mechanism. A related question is the integration between a prosthetic device and additional motion as an augmentation to its use. Suppose that you have a prosthetic left hand, while your right hand is intact, and then wear the motion induction system. Does the adaptation effect in both hands follow a similar or different profile? This is an open question for future research.

*Sense of Agency (SoA)*: Gallagher defines SoA as “[the] sense that I am the one who is causing or generating an action” [28 p. 15]. Blackemore et al. proposed a comparator model, in which a sensory discrepancy between the predicted and actual sensory feedback results in a lack of SoA and accordingly creates disturbances in motor control [7] (Fig 2a). In other words, SoA is considered to directly reflect the dynamics of the motor control system, and perceptual simultaneity plays a major role in the generation of SoA. Indeed, some have used perceptual simultaneity or timing perception as an indicator of agency (i.e., intentional binding), although the relationship between perceptual simultaneity and SoA is situation-dependent [29, 30]. In our case, in all trials, participants’ arm movements were intentional, and the stimulation physically moved their arms at about the same time as their intention to move. So, even when the participant perceived asynchrony, they could still have SoA (i.e., a “judgment of agency” [31]). Kasahara et al. developed a device that actuates the human body with EMS preceding human movement by 80 ms without fully compromising the user’s sense of agency [32]. Furthermore, the relationship between temporal recalibration and SoA has been discussed [33, 34]. Since the management of SoA during motion induction is a critical issue, we will need to design new experiments to assess it in future research.

#### Potential applications

*Application to muscle training*: One of the most straightforward applications is muscle training with EMS. EMS randomly applied or applied with unpredictable timing often results in discomfort. However, it is known that uncomfortable sensations are reduced when the timing of intentional movement and the sensation coincide [6, 35]. Therefore, EMG-triggered EMS muscle training that maintains the perceptual simultaneity between the person’s intention and the EMS trigger is expected to make the training more comfortable.

*Fall prevention for elderly people*: When EMG and EMS technology becomes more compact and portable, a greater diversity of applications can be explored as novel solutions to pressing societal problems. Supporting a growing and longer-lived elderly population, for instance, has become a worldwide challenge in need of innovative approaches, particularly those that consider the agency and dignity of the individual [36]. Fall prevention, for example, remains a pressing healthcare challenge [37]. We can imagine how a discreet, wearable version of our prototype could detect the start of fall motions and help elders respond with a greater sense of agency and confidence.

## Conclusions

In this study, we evaluated an EMG-triggered motion induction system that detects the onset of body movements using an EMG device and stimulates the biceps to induce additional movements through EMS. The results of Experiment 1 provide a DSI range of perceptual simultaneity between intentional and induced movements for future applications. Moreover, the results of Experiment 2 demonstrate that perceptual simultaneity can be shifted depending on the design of the system and series of stimuli. This suggests that the integration between our innate motor control mechanism and induced movements can be experimentally manipulated, to some extent.

We should note that this study only covered the temporal factor of integration and examined a simple discrete movement: arm lifting. As such, this is but a first step towards establishing a human assist system that effectively utilizes the phenomenon of perceptual simultaneity shifting. Further investigations on other factors will surely reveal the mechanism(s) underlying the integration of induced movements with motion intention, leading to a fruitful trajectory of research on human assist systems.

## Supporting information

S1 Fig The distribution of the adaptation delay (Experiment 2). 91% of the system delay in Stim A50 was under 100 ms, indicating that the distributional separation required in this experiment was achieved.
(PDF)

S2 Fig The DSI of each trial shown as a raster plot (Experiment 2). DSI is the interval between detection and stimulation. The circles show the DSI of each trial.
(PDF)
