# Supplementary material for: Perceptual simultaneity and its modulation during EMG-triggered motion induction with electrical muscle stimulation
Source: PLoS One. 2020 Aug 12;15(8):e0236497. doi: 10.1371/journal.pone.0236497 (PMC7423077; doi:10.1371/journal.pone.0236497)
Supplement: S1 Fig — 91% of the system delay in Stim A50 was under 100 ms, indicating that the distributional separation required in this experiment was achieved. (PDF) [file pone.0236497.s001.pdf]

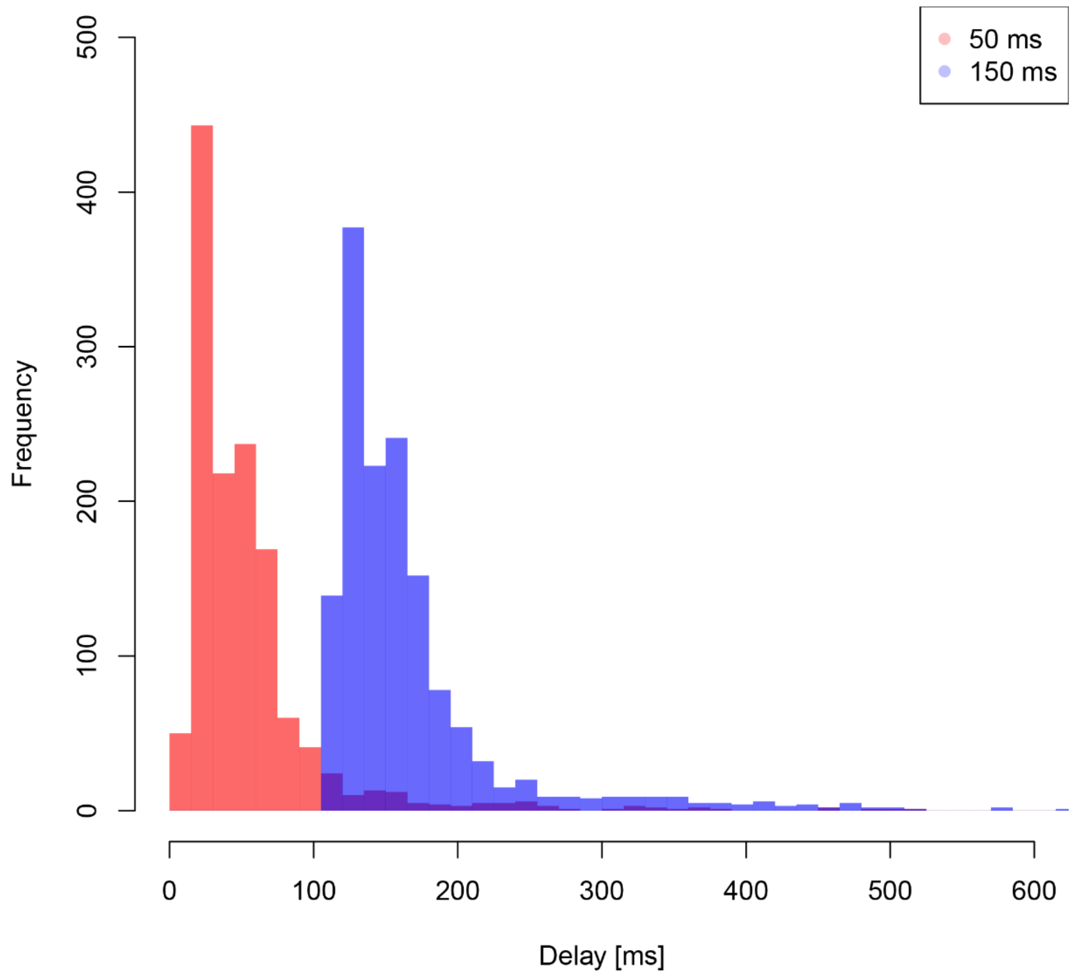

S1 Fig. The distribution of the adaptation delay (Experiment 2). 91% of the system delay in Stim A50 was under 100 ms, indicating that the distributional separation required in this experiment was achieved.
