# Supplementary material for: Perceptual simultaneity and its modulation during EMG-triggered motion induction with electrical muscle stimulation
Source: PLoS One. 2020 Aug 12;15(8):e0236497. doi: 10.1371/journal.pone.0236497 (PMC7423077; doi:10.1371/journal.pone.0236497)
Supplement: S2 Fig — DSI is the interval between detection and stimulation. The circles show the DSI of each trial. (PDF) [file pone.0236497.s002.pdf]

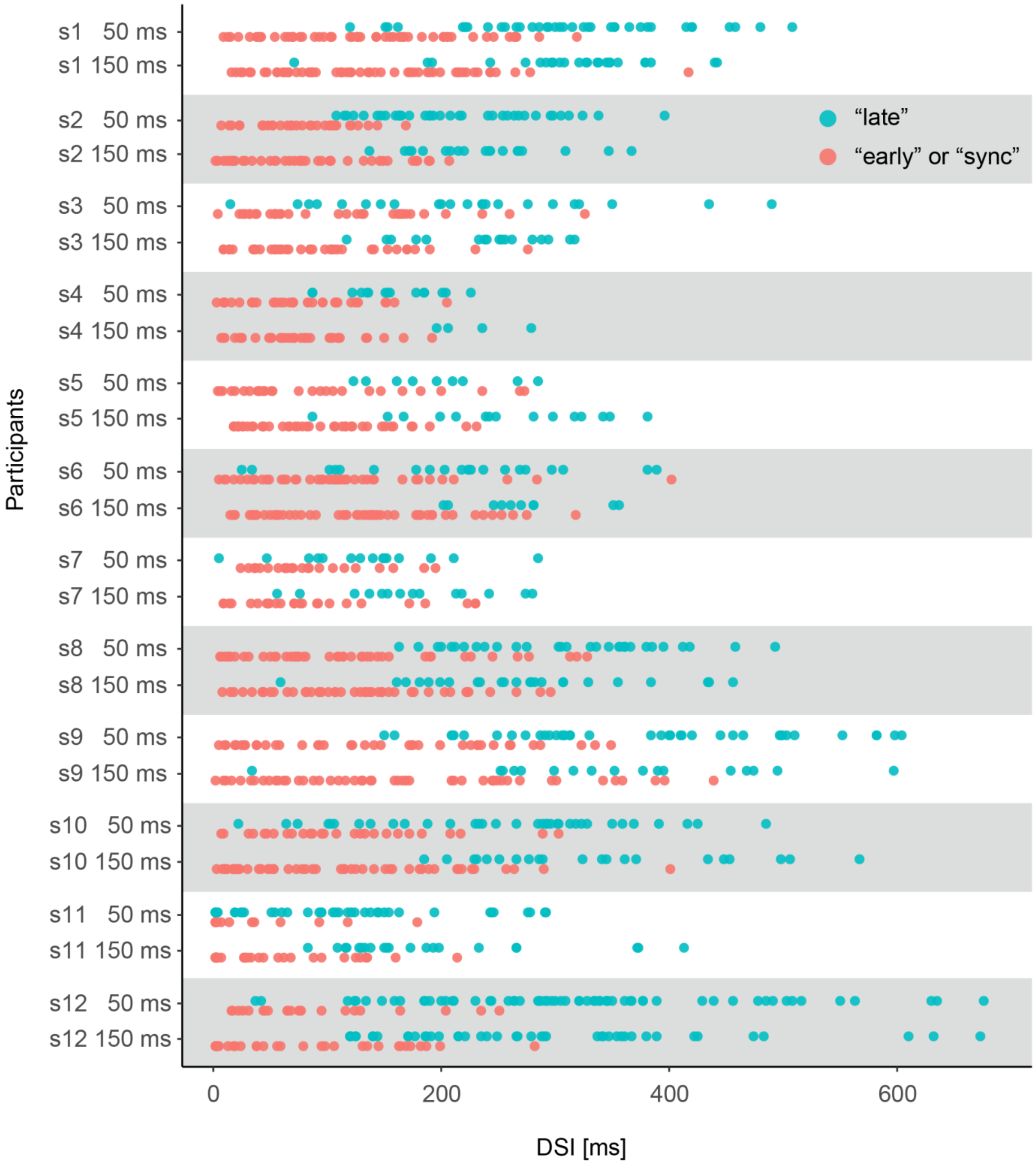

S2 Fig. The DSI of each trial shown as a raster plot (Experiment 2). DSI is the interval between detection and stimulation. The circles show the DSI of each trial.
